# Life-Threatening Adenovirus Infections in the Setting of the Immunocompromised Allogeneic Stem Cell Transplant Patients

**DOI:** 10.1155/2010/601548

**Published:** 2010-06-16

**Authors:** Cedar J. Fowler, Jennifer Dunlap, Darcy Troyer, Peter Stenzel, Elliot Epner, Richard T. Maziarz

**Affiliations:** ^1^Laboratory of Clinical Infectious Diseases—NIAID, National Institutes of Health, 9000 Rockville Pike MSC 1684, Bethesda, MD 20892-1684, USA; ^2^Department of Pathology, Oregon Health and Science University, 3181 SW. Sam Jackson Park Road, Portland, OR 97239, USA; ^3^Adult Blood and Marrow Stem Cell Transplant Program, Oregon Health and Science University, 3181 SW. Sam Jackson Park Road, Portland, OR 97239, USA; ^4^Penn State Hershey Medical Center 500, University Drive, Hershey, PA 17033, USA

## Abstract

A single institution case series of adenovirus infections after allogeneic hematopoietic stem cell transplantation is presented to highlight the consideration for adenovirus infections as an etiology in patients with rapid hepatic or other sudden organ deterioration in the setting of apparent GVHD stabilization. The series also highlights that survival is limited with these infections often due in part to concomitant opportunistic infections. In addition, the pathophysiological events, such as GVHD and hepatic dysfunction, may complicate the clinical picture and delay therapy of an opportunistic infection. This is particularly true for adenoviral infections as they also have a distinct clinical picture in immunocompromised patients when compared to immune competent patients. Adenovirus infections also have the additional challenge that its treatment, cidofovir, has associated toxicities that can delay its administration. Recent developments has yielded an assay that can be used in the early detection and for serial determinations of adenovirus in patients with advanced GVHD, as well as a new therapeutic agent currently undergoing clinical trials.

## 1. Introduction

Inthe majority of immune competent patients, adenovirus infections are often limited to single organ presentation associated with its strong tissue tropism to epithelial surfaces [1, 2]. However in immunocompromised patients, disseminated infections are common and can be difficult to diagnose due to the often associated multiorgan dysfunction arising secondary to the pathophysiological processes that involved the allogeneic transplant [3]. The diagnostic difficulties of identifying this opportunistic infection are magnified in patients who are experiencing multiorgan failure with advanced grade acute graft versus host disease (GVHD), often with concomitant CMV and/or fungal infections [4, 5]. The dilemma in diagnosis of the infection can also be challenging when dealing with complications of hepatic failure secondary to GVHD [6, 7]. As well, in the past due to the lack of adequate diagnostic studies, identification of adenovirus was compromised although it has now improved with molecular screening tools. In this highlighted case history, we provide an illustration of the critical impact that adenovirus infections have on immunocompromised patients with advanced GVHD and propose the recommendation that adenovirus must be considered in patients with rapid hepatic or other sudden organ deterioration in the setting of apparent stabilization of GVHD.

## 2. Case Report and Series

**Figure 1:**
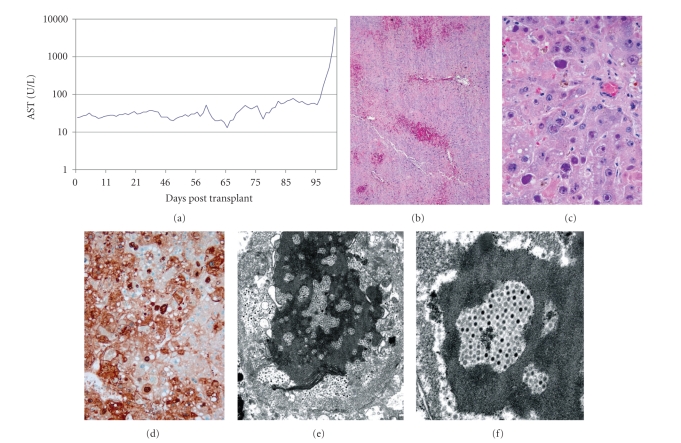
(a) Time course of AST elevation after allogeneic transplant, (b) hepatic necrosis with intraparenchymal hemmorhage (H & E, 4x), (c) hepatocyte nuclei with marked variation in size, with intranuclear inclusions (H & E, 40x), (d) immunohistochemistry stain—positive for adenovirus (40x), (e) electron micrograph of intranuclear adenovirus particles (14,000x), (F) high-resolution electron micrograph image of adenovirus particles (36,000x).

The highlighted patient (number 1 in the following tables) is a 67 years old male with a history of multiply relapsed follicular lymphoma who underwent nonmyeloablative sibling allogenic peripheral blood stem cell transplant. He was admitted on day +44 for suspected acute graft-versus-host disease (aGVHD) with new onset large volume diarrhea with renal insufficiency. The patient proceeded to have a two-month hospital stay notable for incomplete control of grade III GVHD despite interventions with high-dose steroids, tacrolimus, mycophenolate mofetil, and denileukin diftitox. The course was complicated by coagulase-negative Staphylococcus bacteremia, CMV reactivation, and suspected Aspergillus pneumonia requiring antibiotics, ganciclovir, and antifungal therapy, respectively. During a period of apparent convalescence, the patient developed rapid onset of hepatic failure. Differential diagnosis included infection, medication toxicity, and hepatic GVHD. Intervention was futile and the patient succumbed in five days, during which, the AST and ALT increased from 80 and 137 U/L to 6163 and 3626 U/L, respectively, (Figure 1(a)). 

 Autopsy results revealed acute hepatic necrosis secondary to disseminated adenovirus infection and also demonstrated coinfection with Aspergillus nodules identified within the lungs (Figures 1(b) and 1(c)). Immunohistochemical staining for adenovirus was positive in the lamina propria of the small and large intestine, liver, and lung (Figure 1(d)) with confirmation by electron microscopy of the high viral load (Figures 1(e) and 1(f)). In retrospect, the clinical time course was suggestive of infection with adenovirus [4], although initial clinical impressions were that the patient was developing rapidly progressive hepatic GVHD. Interestingly, at the time of autopsy, a two-state (Oregon and Washington) public health advisory warning regarding severe respiratory infection due to adenovirus, in particular serotype 14, was issued [8]. Confirmation of the serotype in this patient was not possible but concerns were raised regarding hospital acquired adenoviral infection. 

 With these considerations,we reviewed all other adenovirus infections experienced by patients undergoing allogeneic transplants in our center in the past five years (Tables 1 and 2). All six patients, including patient 1 described above, had been heavily pretreated prior to proceeding to their allogeneic transplantation procedure. Five of the six patients underwent conventional conditioning with a three-drug GVHD prophylaxis schedule. Five patients developed GVHD early in their treatment course. Notably, the highlighted patient received an unrelated allogeneic reduced intensity transplantation procedure with cyclosporine and mycophenolate mofetil for GVHD prophylaxis, with acute GVHD presenting a little over one week from the scheduled taper of the mycophenolate mofetil. In all circumstances, the subsequent hospital course was complicated by multiple infections and protracted management of steroid resistant acute GVHD and in two of the patients, nosocomial transmission was suspected.

## 3. Discussion

In the majority of these allogeneic stem cell transplant patients, the presentation of adenovirus was late in the GVHD course with fulminant presentation noted in our sentinel patient as well as in two other individuals with respiratory failure. Three patients survived the adenovirus infection although all required very protracted hospital courses, one subsequently died of complications of GVHD and in another of these patients, cidofovir-associated encephalopathy became the major complicating condition. Dosing schedules were altered in three of the patients due to nephrotoxicity issues. Successful clearance of adenovirus was only identified in one patient while in the two surviving patients, adenovirus remained persistently detectable in urine samples consistent with chronic viral cystitis. In all cases, adenovirus was difficult to diagnose due to the complexity of the multiorgan disease processes seen in these allogeneic transplant patients. The presenting symptoms were often mimicked by other infections or by clinically active acute GVHD [9]. The only survivors to date had been detected relatively early in the viral presentation but still experienced severe morbidity. One patient subsequently died of complications of chronic GVHD and the other two survivors currently have an unclear future. Notably, in some of these patients, nosocomial transmission was considered as a possible source. In patient 1, the virus infection coincided with a community outbreak, although it never was determined if his infection was of the same viral strain. In patients 2 and 6, viral infection was identified after patient 4 had experienced prolonged hospitalization for her original presentation with adenovirus cystitis. As a consequence, we feel aggressive infection control isolation procedures with droplet and bodily fluid isolation should be implemented in all patients diagnosed with adenovirus and have implemented this policy. 

What clearly has been beneficial is the development and access to sensitive quantitative PCR assays that were used in the early detection and for serial determinations of adenovirus in these patients with advanced GVHD. New algorithms for utilization of these extremely sensitive molecular tools in this allogeneic transplant setting as screening tools may prove to be critical in altering the natural history of disseminated adenovirus in this immune-suppressed patient population [10–12].

Treatment for adenovirus in hematopoietic stem cell transplant patients is challenging for multiple reasons. As discussed above, diagnosis may be delayed due to confounding picture with GVHD and/or coinfections. Once adenovirus is suspected either by clinical history or non confirmatory diagnostic test, often the treatment with cidofovir is delayed until confirmatory tests have been done due to reluctance of clinicians to use the drug due to its severe side effect profile including renal toxicity and CNS side effects. Extrapolation of these data would suggest that if there was a less toxic drug for the treatment of adenovirus, utilization would be more likely to occur earlier in the infectious time course, thereby decreasing morbidity and mortality. 

 Currently, a new agent that meets this description is being assessed in phase 2 clinical trials across the country. CMX001 is an oral lipid conjugate of cidofovir [13]. This medication has been shown to have effectiveness against all double stranded DNA viruses which includes herpesviruses (CMV, HSV-1 and -2, VZV, EBV, HHV-6, and HHV-8), adenoviruses (AdV3, AdV5, AdV7, AdV8, and AdV31), polyomaviruses (BKV and JC virus), orthopoxviruses (variola, monkeypox, vaccinia, cowpox, and ectromelia viruses), and papillomaviruses (HPV). Advantages include potent activity against all dsDNA viruses, good oral bioavailability, potential safety given decreased dosing and increased potency, and lack of concentration in the kidney epithelial tubule cells. As this case series reflects, a medication that can be used safely for the treatment of localized and/or disseminated adenovirus will be a major advancement; however the GI toxicity often associated with acute GVHD may compromise the oral absorption of the medication. If the promise of this agent materializes without demonstration of a serious side effect profile, one can even imagine institution of the agent as prophylaxis against these viruses and could prove to be of benefit in the hematopoietic stem cell transplant patient population.

## Figures and Tables

**Table 1:** Patient Demographics. Cy/TBI: Cyclophosphamide/Total Body Irradiation, PBSCT: Peripheral Blood Stem Cell Transplant, MUD: Matched unrelated donor, CML: Chronic Myeloid Leukemia, ALL: Acute Lymphocytic Leukemia, CML: Chronic Myeloid Leukemia, M: male, F: female.

	Age/Gender	Disease	Pretransplant Therapy	Conditioning Therapy	Type of Transplant	GVHD Prophylaxis	GVHD Onset	Max Grade GVHD
1	67/M	Follicular Lymphoma	R/CHOP R/DHAP Fludarabine	Busulfan Fludarabine TBI	Allogeneic PBSCT	MMF Cyclosporine	Day +38 Skin, gut Stage II	III
2	44/M	Follicular Lymphoma	R/CHOP FND-R Rituxan + Bortezomib Genmab Anti-CD20 Ab Study R-ICE salvage	Cy/TBI	Sibling Allogeneic PBSCT	Steroids Methotrexate	Day +20 gut and liver Stage III	IV
3	39/M	CML with T315I mutation	Dasatinib Imatinib 3 + 7 HIDAC 3 + 7	Cy/TBI	MUD PBSCT	Steroids Methotrexate Cyclosporine	Day +19 Skin, gut, and conjunctivae Stage IV	IV
4	57/F	Follicular Lymphoma	R/CHOP RICE RESHAP RICE	Cy/TBI	Allogeneic PBSCT	Steroids Methotrexate Cyclosporine	Day +30 Gut Stage II	III
5	42/F	ALL	Hyper CVAD	Cy/TBI	MUD	Steroids Methotrexate Cyclosporine	Day +360 Skin, gut Stage IV	IV
6	49/M	Multiple Myeloma	Local Radiation Lenalidomide Bortezomib/Doxil/Dex	Carmustine Etoposide Cytarabine Mephalan	Allogeneic PBSCT	Steroids Methotrexate Cyclosporine	Day +26 gut Stage II	III

**Table 2:** Clinical course of Adenovirus infections.

	Clinical Presentation	Diagnosis of Adenovirus	Therapy for Adenovirus	Viral Outcome (copies/ml)	Current Status
1	Hepatitis Day +98	Autopsy	None	Death	Death Day +101
2	Pulmonary Day +180	Serum* and BAL PCR	Cidofovir 1mg/kg QOD ×7 doses IVIG ×1 dose	45,000→ 2,000→ negative	ECOG 4 Day +392
3	Pulmonary Day +64	Serum* PCR	Cidofovir 4mg/kg qweek ×3 doses	560,000→ 25,700→ death prior to repeat	Death Day +92
4	Cystitis Day +168	Serum, CSF, Urine* PCR	Cidofovir 1mg/kg QOD ×3 doses IVIG ×2 doses	2 mill→ 15 mill → 800→ no repeat	ECOG 1 Day +434
5	Cystitis Day +218	Urine*, and Serum PCR	Cidofovir 1mg/kg QOD ×7 doses	370,000→ 1,000→ no repeat	Death Day +1020
6	Pulmonary Day +30	Serum*, BAL, and Stool PCR	Cidofovir 1mg/kg QOD ×5 doses IVIG ×3	18,000→ death prior to repeat	Death Day +46

*used to determine viral outcome.
